# HCoV-NL63 and SARS-CoV-2 Share Recognized Epitopes by the Humoral Response in Sera of People Collected Pre- and during CoV-2 Pandemic

**DOI:** 10.3390/microorganisms8121993

**Published:** 2020-12-14

**Authors:** Elena Rita Simula, Maria Antonietta Manca, Seyedsomaye Jasemi, Sergio Uzzau, Salvatore Rubino, Pierangela Manchia, Angela Bitti, Mario Palermo, Leonardo A. Sechi

**Affiliations:** 1 Department of Biomedical Sciences, Division of Microbiology and Virology, University of Sassari, 07100 Sassari, Italy; simulaelena@gmail.com (E.R.S.); m.anto.manca@gmail.com (M.A.M.); somayejasemy@yahoo.com (S.J.); uzzau@uniss.it (S.U.); rubino@uniss.it (S.R.); 2 Patologia Clinica, ATS Sardegna, ASSL, 07100 Sassari, Italy; Pierangela.manchia@aousassari.it (P.M.); angela.bitti@aousassari.it (A.B.); 3 Servizio di Endocrinologia, Azienda Ospedaliera Universitaria (AOU), 07100 Sassari, Italy; mario.palermo@aousassari.it

**Keywords:** HCoV-NL63, SARS-CoV-2, epitopes, antibodies, competitive ELISA

## Abstract

Severe acute respiratory syndrome coronavirus 2 (SARS-CoV-2) can cause serious illness in older adults and people with chronic underlying medical conditions; however, children and young people are often asymptomatic or with mild symptoms. We evaluated the presence of specific antibodies (Abs) response against Human coronavirus NL63 (HCoV-NL63) S protein epitopes (NL63-RBM1, NL63-RBM2_1, NL63-RBM2_2, NL63-RBM3, NL63-SPIKE_541–554_, and NL63-DISC-like) and SARS-CoV-2 epitopes (COV2-SPIKE_421–434_ and COV2-SPIKE_742–759_) in plasma samples of pre-pandemic, mid-pandemic, and COVID-19 cohorts by indirect ELISA. Moreover, a competitive assay was performed to check for cross reactivity response between COV2-SPIKE_421–434_ and NL63-RBM3 among patients with a definitive diagnosis of SARS-CoV-2. Immune reaction against all SARS-CoV-2 and HCoV-NL63 epitopes showed a significantly higher response in pre-pandemic patients compared to mid-pandemic patients. The results indicate that probably antibodies against HCoV-NL63 may be able to cross react with SARS-CoV-2 epitopes and the higher incidence in pre-pandemic was probably due to the timing of collection when a high incidence of HCoV-NL63 is reported. In addition, the competitive assay showed cross-reactivity between antibodies directed against COV2-SPIKE_421–434_ and NL63-RBM3 peptides. Pre-existing HCoV-NL63 antibody response cross reacting with SARS-CoV-2 has been detected in both pre- and mid-pandemic individual, suggesting that previous exposure to HCoV-NL63 epitopes may produce antibodies which could confer a protective immunity against SARS-CoV-2 and probably reduce the severity of the disease.

## 1. Introduction 

Coronaviruses (CoVs) are the largest identified family of RNA viruses with positive-sense, non-segmented single-stranded RNA genome (26–32 kilobases in size) [1]. Nowadays, human CoVs (HCoVs) belong to two groups: alpha-CoV which includes HCoV-NL63, HCoV-229E, and beta-CoV comprised of HCoV-OC43, HCoV-HKU1, SARS-CoV, MERS-CoV, and SARS-CoV-2 [2]. HCoV-NL63, HCoV-229E, HCoV-OC43, and HCoV-HKU1 cause mainly mild infections of the upper respiratory tract, while SARS-CoV, MERS-CoV, and SARS-CoV-2 are responsible for severe disease with high morbidity and mortality rate [3].

The novel coronavirus disease 2019 (COVID19) was first reported in Wuhan, China, in December 2019, and declared a worldwide pandemic on 11 March 2020 (https://www.ecdc.europa.eu/en/novel-coronavirus/event-background-2019). As of 12 December 2020, Johns Hopkins, University of Medicine, has reported 71,081,574 confirmed cases and 1,594,777 death cases worldwide [4]. Older adults and people with chronic underlying medical conditions are at higher risk for serious illness from SARS-CoV-2; however, children represent a small portion of patients whose clinical symptoms are usually milder than adults [5]. There has been multiple evidence about reduced susceptibility in children, probably due to cross-protection from previous CoVs infections [6,7].

SARS-CoV-2, as well as HCoV-NL63, interact with host cell by the envelope protein spike (S) required for the binding with angiotensin converting enzyme 2 (ACE2) receptor through Receptor Binding Domain (RBD) and for membrane fusion [8,9,10], these first steps point out to be crucial for the beginning of the infection [11].

RBD contains Receptor-Binding Motifs (RBMs) capable of interacting with ACE2 [12] and prompting the synthesis of neutralizing antibodies thanks to the presence of epitopes with immunogenic properties [13]. In regard to what mentioned above, the infusion of convalescent plasma (CP) from recovered COVID19 subjects into ongoing diseased patients seems to protect from the severity of the infection [14].

Different studies evidenced a certain degree of antigenic cross-protection for human CoVs belonging to the same genetic group, such as the protection offered against HcoV-229E by HcoV-NL63 seroconversion (both alpha-coronaviruses) or the protection against HcoV-HKU1 reinfection related to HcoV-OC43 seroconversion [15]. Seroprevalence surveys pinpoint that children are seropositive to HcoV-NL63 by six years old [16] and that reinfection by the same virus occurs throughout life, keeping in mind that nearly 20% of clinical cold can be accounted to four different genotypes of CoVs. Given the lack of symptoms in the majority of pediatric patients during SARS-CoV-2 infection and the capability of both SARS-CoV-2 and HcoV-NL63 S-protein to bind ACE2, we pondered the possibility that antibodies against HcoV-NL63 may cross react and offer protection against SARS-CoV-2 infection. The Receptor Binding Domain (RBD), within S, is the main target for neutralizing antibodies and was selected as the ideal vaccination subunit against SARS-CoV-2 and previous SARSs. The presence of antibodies against these specific epitopes may correlate with disease outcome and eventually with vaccine efficacy. Different monoclonal antibodies (mAbs) against S protein have been reported [17]. None of the mAbs that were reported bound to prefusion ectodomain trimers of the HcoV-OC43 or MERS-CoV S glycoproteins, which indicated a lack of cross-reactivity outside the Sarbecovirus subgenus.

With the aim to identify cross-reacting antibodies recognizing different coronaviruses, we evaluated the antibody response against different epitopes of SARS-CoV-2 and HcoV-NL63 S-protein in a pre-pandemic, a mid-pandemic, and a SARS-CoV-2 positive cohort.

## 2. Materials and Methods

### 2.1. Subjects and Blood Collection 

Study population was formed by a pre-pandemic COVID19 cohort of 160 patients (109 females and 51 males; mean age ± SD, 39.63 ± 22.85 years), a mid-pandemic COVID19 cohort of 141 patients (105 females and 36 males; mean age ± SD, 55.55 ± 16.99). Both cohorts were recruited from the Endocrinology Department of University Hospital, Sassari; the pre-pandemic group was enrolled during the winter and early spring of 2017 and 2018, while the mid-pandemic one from May to August 2020. Peripheral venous blood samples were collected in K2-EDTA tube test and separated plasma was tested for the presence of antibodies against SARS-CoV-2 and HCoV-NL63 Spike protein-derived peptides. A group of 46 patients with a definitive diagnosis of SARS-CoV-2 determined by swab test viral PCR was tested against SARS-CoV2 epitopes; four of them (1 female and 3 males; mean age ± SD, 72.5 ± 4.36 years) were admitted to the intensive care unit (ICU) and forty-two (25 females and 17 males; mean age ± SD, 56.95 ± 17.46 years) were hospitalized in the SARS-CoV-2 wards, or other clinical departments. The investigation of plasma was approved by the ethical committee of ASL 1 Sassari (2149/CE). (Table 1)

### 2.2. Peptides

Peptides NL63-RBM1, NL63-RBM2_1, NL63-RBM2_2, NL63-RBM3, NL63-SPIKE_541–554_, NL63-DISC-like, COV2-SPIKE_421–434_, and COV2-SPIKE_742–759_ were designed using Immuno epitope database and analysis resource (IEBD) and synthesized at > 95% purity (LifeTein, South Plainfield, NJ, USA). All peptides were dissolved in DMSO and stored at −80 °C in single-use aliquots (10 mM). (Table 2)

### 2.3. Enzyme-Linked Immunosorbent Assay (ELISA)

Indirect ELISA was performed to detect specific antibodies (Abs) against NL63-RBM1, NL63-RBM2_1, NL63-RBM2_2, NL63-RBM3, NL63-SPIKE_541–554_, NL63-DISC-like, COV2-SPIKE_421–434_, and COV2-SPIKE_742–759_ peptides. Ninety-six-well Nunc immunoplates were incubated overnight at 4 °C with a solution 0.05 M of carbonate-bicarbonate, pH 9.5 (Sigma-Aldrich, St. Louis, USA) and the respective peptides at 10 µg/mL. Plates were incubated one hour at room temperature in a blocking solution with 5% non-fat dried milk (Sigma-Aldrich, St. Louis, USA) and phosphate-buffered saline (PBS) and washed twice in a solution with 0.05% Tween-20 and PBS (PBS-T). Plasma samples were added at 1:100 concentration and incubated for two hours. Then each plate was washed five times in PBS-T and incubated one hour at Rt with 100 µL of PBS and alkaline phosphate-conjugated goat anti-human IgG polyclonal antibody (1:1000, Sigma-Aldrich, St. Louis, MI, USA). After another washing step in PBS-T, plates were incubated in a dark environment for eight to ten minutes in milli-Q water and *p*-nytrophenyl phosphate (Sigma-Aldrich, St. Louis, MI, USA) and the absorbance was read at 405 nm using SpectraMax Plus 384 microplate reader (Molecular Devices, Sunnyvale, CA, USA). All samples were repeated in duplicated and positive controls were used for each peptide.

The obtained absorbance values (at 405 nm) were normalized to a highly positive control serum with absorbance reactivity set at 1.0 arbitrary units (AU/mL).

### 2.4. Rapid Test

nCoV-K003: SARS-CoV-2-IgM-IgG combined rapid test kit (Bio Bench) was used to confirm the presence of IgG and IgM antibodies against SARS-CoV-2 in selected pre-pandemic, co pandemic sample and COVID-19 patients. 10 uLof plasma sample diluted in 60 μL of PBS (pH 7.2) was incubated for 15 min before the reading the result.

### 2.5. Competitive ELISA

Plasma from 11 COVID19 patients, with strong and medium antibody response against the COV2-SPIKE_421–434_ peptide, were incubated on PBS-T (1:100) over night at 4 °C with NL63-RBM3 at concentrations of 10 μM to perform the competitive assay (cELISA).

Treated COVID19 plasma was then subjected to ELISA on plates coated with COV2-SPIKE_421–434_. In the same assay, peptide without the plasma was used as a negative control, COV2-SPIKE_421–434_ positive plasma was used as positive control.

### 2.6. Statistical Analysis

All data were analyzed using GraphPad Prism 8.2.0 software (GraphPad Software, San Diego, CA, USA). Mann–Whitney U and Kruskal–Wallis tests were used to analyze non-parametric data and compare differences between two or three groups, respectively. Differences with a *p*-value < 0.05 were considered statistically significant. Receiver-operating characteristic (ROC) was used to choose the cut-off value to assess the sample positivity, which was consequently tested through Fisher’s exact test. Spearman Correlation tests were performed between HCoV-NL63 and SARS-CoV2-derived peptides.

## 3. Results

Humoral response against five selected epitopes of HCoV-NL63 and two epitopes of SARS-CoV-2 were evaluated in plasma of pre-pandemic, mid-pandemic, and SARS-CoV-2 positive groups.

**Figure 1:**
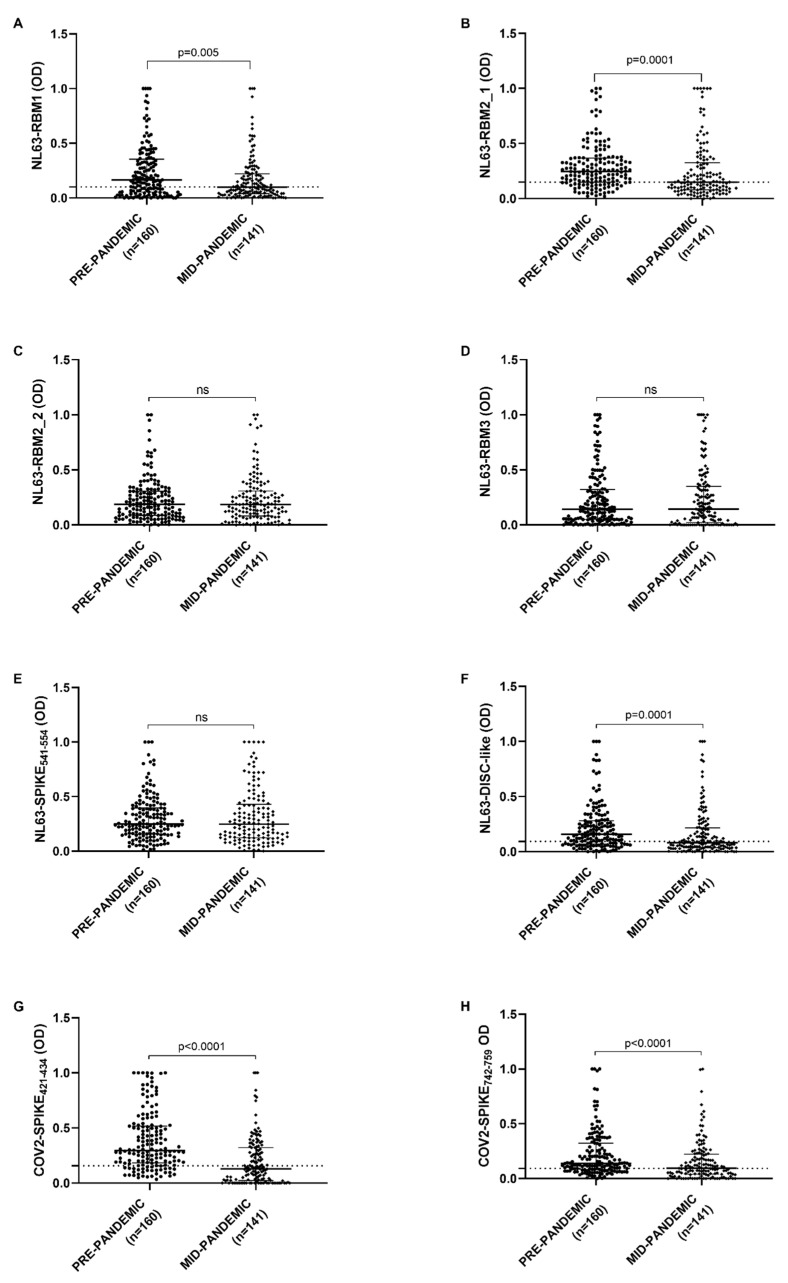
ELISA-based analysis of Abs reactivity against SARS-CoV-2 and HCoV-NL63 derived peptides. Plasma samples were tested against NL63-RBM1 (**A**), NL63-RBM2_1 (**B**), NL63-RBM2_2 (**C**), NL63-RBM3 (**D**), NL63-SPIKE_541–554_ (**E**), NL63-DISC-like (**F**), COV2-SPIKE_421–434_ (**G**), and COV2-SPIKE_742–759_ (**H**) peptides. Scatter plots represent median with interquartile range. Dashed lines represent thresholds used to assess the samples positivity.

Antibody response against NL63-RBM1, showed a statistical difference between pre-pandemic and mid-pandemic populations (*p* = 0.0055) analyzed by Mann–Whitney U test. A cut-off values were chosen by ROC analysis and percentage of positive and negative samples were determined by Fisher’s exact test. 63.1% of pre-pandemic population (101 patients out of 160) and 48.9% in mid-pandemic population (69 patients out of 141) were seropositive (*p* = 0.01, Fisher’s exact test), with a cut-off value of 0.100 and AUC = 0.593 (Figure 1A).

NL63-DISC-like and NL63-RBM2_1 antibody response revealed a statistical difference between pre-pandemic and mid-pandemic population both with *p* = 0.0001. 112 pre-pandemic patients out of 160 (70%) and 69 mid-pandemic patients out of 141 were positive (48.9%) (*p* = 0.0002, Fisher’s exact test) with a cut-off value of 0.087, AUC = 0.627 and *p* = 0.0001 for NL63-DISC-like (Figure 1F); while 120 pre-pandemic patients out of 160 (75%) and 69 mid-pandemic patients out of 141 were positive (48.9%) for NL63-RBM2_1 (*p* < 0.0001, Fisher’s exact test) with a cut-off value of 0.149, AUC = 0.630 and *p* = 0.0001 (Figure 1B,F).

Concerning COV2-SPIKE_421–434_ and COV2-SPIKE_742–759_, the antibody response were significantly different between pre-pandemic and mid-pandemic population, both with a *p* < 0.0001 (Figure 1G,H). The analysis of COV2-SPIKE_421–434_ peptide shows 128 positive patients out of 160 (80%) in pre-pandemic population and 60 positive patients out of 141 (42.5%) in mid-pandemic population (*p* < 0.0001, Fisher’s exact test) with a cut-off value of 0.157, AUC = 0.736 and *p* < 0.0001; about COV2-SPIKE_742–759_, 111 patients out of 160 were positive (69.4%) and 72 patients out of 141 (51.1%) respectively in pre-pandemic and mid-pandemic population (*p* = 0.001, Fisher’s exact test) with a cut-off value of 0.093, AUC = 0.633 and *p* < 0.0001 (Figure 1G,H).

Antibody response against NL63-RBM2_2, NL63-RBM3, and NL63-SPIKE_541–554_ were not significantly different between the two populations (Figure 1C–E).

Correlations analysis were performed on the basis of OD values obtained from evaluation of humoral response against HCoV-NL63 and SARS-CoV-2 epitopes. Strikingly, in pre-pandemic COVID19 group, the antibody response against SARS-CoV-2 epitopes revealed a positive correlation with the antibody response to NL63-derived peptides.

**Figure 2:**
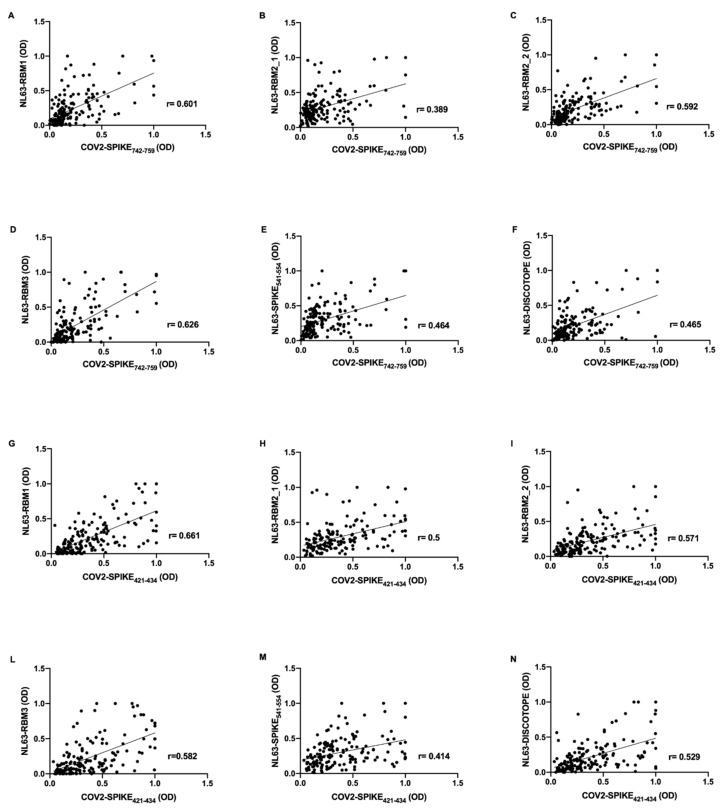
Scatter plot expressing correlation between SARS-CoV-2 and HCoV-NL63 derived peptides in pre-pandemic COVID19 cohort. The graphs show the correlation between COV2-SPIKE_742–759_ and NL63-RBM1 (**A**), NL63-RBM2_1 (**B**), NL63-RBM2_2 (**C**), NL63-RBM3 (**D**), NL63-SPIKE_541–554_ (**E**), NL63-DISC-like (**F**), and between COV2-SPIKE_421–434_ and NL63-RBM1 (**G**), NL63-RBM2_1 (**H**), NL63-RBM2_2 (**I**), NL63-RBM3 (**L**), NL63-SPIKE_541–554_ (**M**), and NL63-DISC-like (**N**).

In particular, COV2-SPIKE_742–759_ presents moderate correlation with NL63-RBM1 (*r* = 0.601, *p* < 0.0001) (Figure 2A), NL63-RBM2_2 (*r* = 0.592, *p* < 0.0001) (Figure 2C), NL63-RBM3 (*r* = 0.626, *p* < 0.0001) (Figure 2D); low correlation has been observed for NL63-RBM2_1 (*r* = 0.389, *p* < 0.0001) (Figure 2B), NL63-SPIKE_541–554_ (*r* = 0.464, *p* < 0.0001) (Figure 2E), and NL63-DISC-like (*r* = 0.465, *p* < 0.0001) (Figure 2F).

Moderate correlations were observed also between COV2-SPIKE_421–434_ and NL63-RBM1 (*r* = 0.661, *p* < 0.0001) (Figure 2G), NL63-RBM2_1 (*r* = 0.5, *p* < 0.0001) (Figure 2H), NL63-RBM2_2 (*r* = 0.571, *p* < 0.0001) (Figure 2I), NL63-RBM3 (*r* = 0.582, *p* < 0.0001) (Figure 2L), and NL63-DISC-like (*r* = 0.529, *p* < 0.0001) (Figure 2N); low correlation was noticed between COV2-SPIKE_421–434_ and NL63-SPIKE_541–554_ (*r* = 0.414, *p* < 0.0001) (Figure 2M).

**Figure 3:**
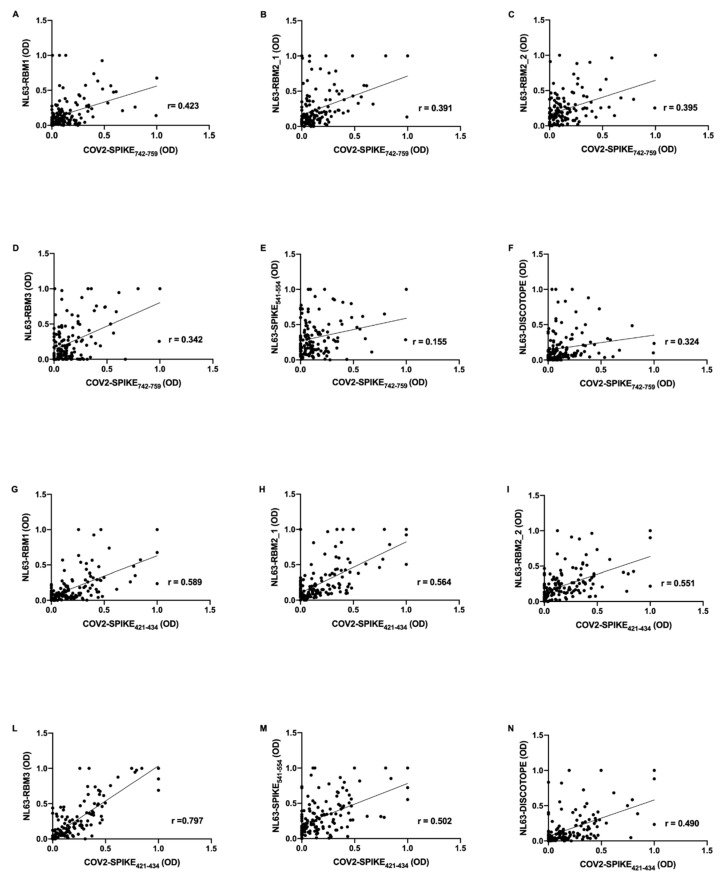
Scatter plot expressing correlation between SARS-CoV-2 and HCoV-NL63 derived peptides in mid-pandemic COVID19 cohort. The graphs show the correlation between COV2-SPIKE_742–759_ and NL63-RBM1 (**A**), NL63-RBM2_1 (**B**), NL63-RBM2_2 (**C**), NL63-RBM3 (**D**), NL63-SPIKE_541–554_ (**E**), NL63-DISC-like (**F**), and between COV2-SPIKE_421–434_ and NL63-RBM1 (**G**), NL63-RBM2_1 (**H**), NL63-RBM2_2 (**I**), NL63-RBM3 (**L**), NL63-SPIKE_541–554_ (**M**), NL63-DISC-like (**N**).

Further, mid-pandemic group’s antibody response presents positive correlations between SARS-CoV-2 epitopes and NL63 derived peptides. COV2-SPIKE_742–759_ exhibit low correlation with NL63-RBM1 (*r* = 0.423, *p* < 0.0001) (Figure 3A), NL63-RBM2_1 (*r* = 0.391, *p* < 0.0001) (Figure 3B), NL63-RBM2_2 (*r* = 0.395, *p* < 0.0001) (Figure 3C), NL63-RBM3 (*r* = 0.342, *p* < 0.0001) (Figure 3D), NL63-DISC-like (*r* = 0.324, *p* = 0.0001) (Figure 3F), and a very low correlation with NL63-SPIKE_541–554_ (*r* = 0.155, *p* = ns) (Figure 3E).

COV2-SPIKE_421–434_ showed a moderate correlation with NL63-RBM1 (*r* = 0.589, *p* < 0.0001) (Figure 3G), NL63-RBM2_1 (*r* = 0.564, *p* < 0.0001) (Figure 3H), NL63-RBM2_2 (*r* = 0.551, *p* < 0.0001) (Figure 3I), NL63-SPIKE_541–554_ (*r* = 0.502, *p* < 0.0001) (Figure 3M), and a low correlation with NL63-DISC-like (*r* = 0.490, *p* < 0.0001) (Figure 3N). A remarkable high correlation was observed between COV2-SPIKE_421–434_ and NL63-RBM3 (*r* = 0.797, *p* < 0.0001) (Figure 3L).

**Figure 4:**
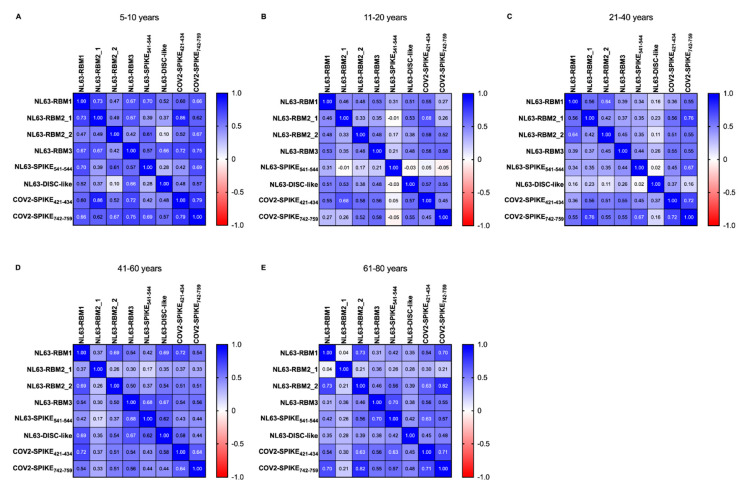
Heatmap displaying the r values obtained from Spearman correlation analysis performed among HCoV-NL63 and SARS-CoV-2 derived peptides used in the previous experiments in the pre-pandemic cohort divided by age groups (**A**) (5–10 years old), (**B**) (11–20 years old), (**C**) (21–40 years old), (**D**) 41–60 years/old) and (**E**) (61–80 years old), as shown in Table 3.

In order to study the age-variability of the pre-pandemic population, we performed a correlation analysis between HCoV-NL63 and SARS-CoV-2 epitopes, calculated using Spearman correlation test. The heatmaps (Figure 4) show *r*’s value between pairs of epitopes of HCoV-NL63 and SARS-CoV-2 in pre-pandemic population divided by age groups, as shown in Table 3.

In the 5–10 years group (Figure 4A) we have not observed statistical difference between NL63-RBM1 and NL63-RBM2_2, NL63-RBM1 and NL63-DISC-like, NL63-RBM2_1 and NL63-DISC-like, NL63-RBM2_2 and NL63-RBM2_1, NL63-RBM1 and NL63-RBM2_2, NL63-RBM2_1 and NL63-SPIKE_541–554_, NL63-RBM2_2 and NL63-RBM3, NL63-RBM2_2 and NL63-DISC-like, NL63-RBM2_2 and COV2-SPIKE_421–434_, NL63-SPIKE_541–554_ and NL63-DISC-like, NL63-SPIKE_541–554_ and COV2-SPIKE_421–434_, and lastly between NL63-DISC-like and COV2-SPIKE_421–434_. High correlations are detectable between NL63-RBM1 and NL63-RBM2_1 (*r* = 0.735, *p* = 0.004), NL63-RBM2_1 and COV2-SPIKE_421–434_ (*r* = 0.859, *p* < 0.0001), NL63-RBM3 and COV2-SPIKE_421–434_ (*r* = 0.724, *p* = 0.005), NL63-RBM3 and COV2-SPIKE_742–759_ (*r* = 0.745, *p* = 0.003), COV2-SPIKE_421–434_ and COV2-SPIKE_742–759_ (*r* = 0.79, *p* = 0.001). Moderate correlations exist among NL63-RBM1 and the epitopes NL63-RBM3 (*r* = 0.675, *p* = 0.01), NL63-SPIKE_541–554_ (*r* = 0.697, *p* = 0.007), COV2-SPIKE_421–434_ (*r* = 0.596, *p* = 0.027), COV2-SPIKE_742–759_ (*r* = 0.662, *p* = 0.012). NL63-RBM2_1 shows moderate correlation with NL63-RBM3 (*r* = 0.667, *p* = 0.011), COV2-SPIKE_742–759_ (*r* = 0.623, *p* = 0.02); same level of correlation was observed also between NL63-RBM2_2 and NL63-SPIKE_541–554_ (*r* = 0.614, *p* = 0.022), NL63-RBM2_2 and COV2-SPIKE_742–759_ (*r* = 0.67, *p* = 0.011), NL63-RBM3 and NL63-SPIKE_541–554_ (*r* = 0.572, *p* = 0.035), NL63-RBM3 and NL63-DISC-like (*r* = 0.657, *p* = 0.013), COV2-SPIKE_742–759_ and NL63-SPIKE_541–554_ (*r* = 0.686, *p* = 0.008), and the between COV2-SPIKE_742–759_ and NL63-DISC-like (*r* = 0.569, *p* = 0.037).

In the 11–20 years group (Figure 4B) a non-significant *p*-value has been found for the correlation between NL63-RBM2_1 and NL63-RBM2_2, NL63-SPIKE_541–554_ and the epitopes NL63-RBM1, NL63-RBM2_1, NL63-RBM2_2 and NL63-RBM3; the correlations were no statistically significant also between NL63-DISC-like and NL63-SPIKE_541–554_, COV2-SPIKE_421–434_ and NL63-SPIKE_541–554_, and for COV2-SPIKE_742–759_ correlated with NL63-RBM1, NL63-RBM2_1 and NL63-SPIKE_541–554_ peptides. Moderate correlations were observed among NL63-RBM3 and NL63-RBM1 (*r* = 0.525, *p* = 0.002), NL63-DISC-like and NL63-RBM1 (*r* = 0.511, *p* = 0.002), COV2-SPIKE_421–434_ and NL63-RBM1 (*r* = 0.552, *p* = 0.0009), NL63-DISC-like and NL63-RBM2_1 (*r* = 0.529, *p* = 0.002), COV2-SPIKE_421–434_ and the epitopes NL63-RBM2_1 (*r* = 0.678, *p* < 0.0001), NL63-RBM2_2 (*r* = 0.580, *p* = 0.0004), NL63-RBM3 (*r* = 0.560, *p* = 0.0007), NL63-DISC-like (*r* = 0.573, *p* < 0.0001); a moderate correlation was also found between COV2-SPIKE_742–759_ and the epitopes RBM2_2 (*r* = 0.520, *p* = 0.002), NL63-RBM3 (*r* = 0.579, *p* < 0.0001) and NL63-DISC-like (*r* = 0.553, *p* = 0.001). Low correlations are present between NL63-RBM2_2 and NL63-RBM1 (*r* = 0.479, *p* = 0.005), NL63-RBM2_1 and NL63-RBM1 (*r* = 0.458, *p* < 0.007), NL63-RBM3 and NL63-RBM2_1 (*r* = 0.351, *p* = 0.04), NL63-RBM3 and NL63-RBM2_2 (*r* = 0.481, *p* = 0.005), NL63-DISC-like and NL63-RBM2_2 (*r* = 0.377, *p* = 0.03), NL63-DISC-like and NL63-RBM3 (*r* = 0.478, *p* = 0.005) and finally between COV2-SPIKE_742–759_ and COV2-SPIKE_421–434_ (*r* = 0.448, *p* = 0.009).

In the 21–40 years group (Figure 4C) the correlations were not statistically significant between NL63-DISC-like and COV2-SPIKE_742–759_, COV2-SPIKE_421–434_ and NL63-DISC-like, COV2-SPIKE_421–434_ and NL63-RBM1, NL63-RBM3 and NL63-RBM1, NL63-RBM3 and NL63-RBM2_1, NL63-SPIKE_541–554_ and the epitopes NL63-RBM1, NL63-RBM2_1 and NL63-RBM2_2; no statistically significant correlations were identified between NL63-DISC-like and the epitopes NL63-RBM1, NL63-RBM2_1, NL63-RBM2_2, NL63-RBM3 and NL63-SPIKE_541–554_. High correlations were observed between NL63-RBM1 and COV2-SPIKE_742–759_ (*r* = 0.759, *p* < 0.0001), COV2-SPIKE_742–759_ and COV2-SPIKE_421–434_ (*r* = 0.720, *p* < 0.0001). Moderate correlations were observed between NL63-RBM2_1 and NL63-RBM1 (*r* = 0.562, *p* = 0.003), NL63-RBM2_2 and NL63-RBM1 (*r* = 0.641, *p* = 0.0006), COV2-SPIKE_421–434_ and the epitopes NL63-RBM2_1 (*r* = 0.558, *p* = 0.004), NL63-RBM2_2 (*r* = 0.506, *p* = 0.01), NL63-RBM3 (*r* = 0.547, *p* = 0.005), COV2-SPIKE_742–759_ and the epitopes NL63-RBM1 (*r* = 0.555, *p* = 0.004), NL63-RBM2_1 (*r* = 0.546, *p* = 0.005), NL63-RBM3 (*r* = 0.548, *p* = 0.005), NL63-SPIKE_541–554_ (*r* = 0.670, *p* < 0.0001); low correlation exist between NL63-RBM2_2 and NL63-RBM2_1 (*r* = 0.417, *p* = 0.03), NL63-RBM3 and RBM2_2 (*r* = 0.450, *p* = 0.02), COV2-SPIKE_421–434_ and NL63-SPIKE_541–554_ (*r* = 0.452, *p* = 0.02).

In regard of the 41–60 age group (Figure 4D), the analysis revealed a very low correlation between NL63-SPIKE_541–554_ and NL63-RBM2_1 (*r* = 0.171, *p* = ns), NL63-RBM3 and NL63-RBM2_1 (*r* = 0.295, *p* = 0.03), NL63-RBM2_2 and NL63-RBM2_1 (*r* = 0.258, *p* = ns). A high correlation was obtained between COV2-SPIKE_421–434_ and NL63-RBM1 (*r* = 0.720, *p* < 0.0001), whereas it identified moderate correlations between COV2-SPIKE_421–434_ and NL63-RBM2_2 (*r* = 0.514, *p* = 0.0001), COV2-SPIKE_421–434_ and NL63-RBM3 (*r* = 0.538, *p* < 0.0001), COV2-SPIKE_421–434_ and NL63-DISC-like (*r* = 0.577, *p* < 0.0001), COV2-SPIKE_742–759_ and NL63-RBM1 (*r* = 0.539, *p* < 0.0001), COV2-SPIKE_742–759_ and NL63-RBM2_2 (*r* = 0.508, *p* = 0.0001), COV2-SPIKE_742–759_ and NL63-RBM3 (*r* = 0.565, *p* < 0.0001), but also between COV2-SPIKE_421–434_ and COV2-SPIKE_742–759_ (*r* = 0.640, *p* < 0.0001). Moderate correlations were also found among different peptides recognizing different epitopes of HCoV-NL63 S-protein, particularly NL63-DISC-like and NL63-RBM1 (*r* = 0.689, *p* < 0.0001), NL63-DISC-like and NL63-RBM2_2 (*r* = 0.538, *p* < 0.0001), NL63-DISC-like and NL63-RBM3 (*r* = 0.673, *p* < 0.0001), NL63-DISC-like and NL63-SPIKE_541–554_ (*r* = 0.623, *p* < 0.0001), NL63-RBM3 and NL63-SPIKE_541–554_ (*r* = 0.683, *p* < 0.0001), NL63-RBM3 and NL63-RBM1 (*r* = 0.539, *p* < 0.0001), NL63-RBM2_2 and NL63-RBM1 (*r* = 0.694, *p* < 0.0001). A low correlation has been observed between COV2-SPIKE_742–759_ and NL63-RBM2_1 (*r* = 0.330, *p* = 0.019), COV2-SPIKE_742–759_ and NL63-SPIKE_541–554_ (*r* = 0.442, *p* = 0.001), COV2-SPIKE_742–759_ and NL63-DISC-like (*r* = 0.441, *p* = 0.001), COV2-SPIKE_421–434_ and NL63-RBM2_1 (*r* = 0.366, *p* = 0.009), COV2-SPIKE_421–434_ and NL63-SPIKE_541–554_ (*r* = 0.431, *p* = 0.001), NL63-DISC-like and NL63-RBM2_1 (*r* = 0.348, *p* = 0.01), NL63-SPIKE_541–554_ and NL63-RBM1 (*r* = 0.416, *p* = 0.003), NL63-SPIKE_541–554_ and NL63-RBM2_2 (*r* = 0.369, *p* = 0.008), NL63-RBM3 and NL63-RBM2_2 (*r* = 0.497, *p* = 0.0002), NL63-RBM2_1 and NL63-RBM2_2 (*r* = 0.375, *p* = 0.007).

The last class, namely the 61–80 years group (Figure 4E) presents no statistically significant correlations between NL63-DISC-like and NL63-RBM2_1, COV2-SPIKE_421–434_ and NL63-RBM2_1, COV2-SPIKE_742–759_ and NL63-RBM2_1, NL63-RBM3 and NL63-RBM1, NL63-RBM2_2 and NL63-RBM2_1, NL63-RBM2_1 and NL63-RBM1.

High correlations were observed between NL63-RBM2_2 and NL63-RBM1 (*r* = 0.725, *p* < 0.0001), COV2-SPIKE_742–759_ and the epitopes NL63-RBM1 (*r* = 0.704, *p* < 0.0001), NL63-RBM2_2 (*r* = 0.824, *p* < 0.0001), COV2-SPIKE_421–434_ (*r* = 0.713, *p* < 0.0001); also, NL63-SPIKE_541–554_ and NL63-RBM3 showed a high correlation (*r* = 0.702, *p* < 0.0001). Moderate level of correlation was observed between NL63-SPIKE_541–554_ and NL63-RBM2_2 (*r* = 0.564, *p* = 0.001), COV2-SPIKE_421–434_ and the epitopes NL63-RBM1(r = 0.537, *p* = 0.0009), NL63-RBM2_2 (*r* = 0.627, *p* = 0.0001), NL63-RBM3 (*r* = 0.561, *p* = 0.0005), NL63-SPIKE_541–554_ (*r* = 0.626, *p* = 0.0001); COV2-SPIKE_742–759_ exhibit a moderate correlation with NL63-RBM3 (*r* = 0.546, *p* = 0.0006) and NL63-SPIKE_541–554_ (*r* = 0.574, *p* = 0.0003). Low correlations were found between NL63-RBM3 and the epitopes NL63-RBM2_1 (*r* = 0.356, *p* = 0.03), and NL63-RBM2_2 (*r* = 0.455, *p* = 0.006); NL63-SPIKE_541–554_ and NL63-RBM1 (*r* = 0.425, *p* = 0.01), NL63-DISC-like and the epitopes NL63-RBM1 (*r* = 0.347, *p* = 0.04), NL63-RBM2_2 (*r* = 0.392, *p* = 0.02), NL63-RBM3 (*r* = 0.378, *p* = 0.02), NL63-SPIKE_541–554_ (*r* = 0.420, *p* = 0.01), and for NL63-DISC-like and the epitopes COV2-SPIKE_421–434_ (*r* = 0.45, *p* = 0.007), and COV2-SPIKE_742–759_ (*r* = 0.483, *p* = 0.003).

**Figure 5:**
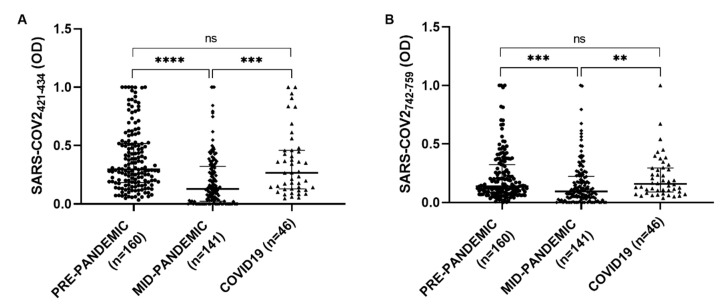
ELISA-based analysis of Abs reactivity against Spike SARS-CoV-2 derived peptides in pre-pandemic, mid-pandemic and COVID19 populations. Plasma samples were tested against COV2-SPIKE_742–759_ (**A**) and COV2-SPIKE_421–434_ (**B**) peptides. Kruskal–Wallis test was used for the analyses. Scatter plots represent median with interquartile range; *p* < 0.0001 (****), *p* < 0.001 (***), *p* < 0.01 (**).

The serological presence of antibodies against COV2-SPIKE_742–759_ and COV2-SPIKE_421–434_ was examined in a group of 46 patients with a diagnosis of SARS-CoV-2, a Kruskal–Wallis test was performed to compare COVID19 group with the pre-pandemic and the mid-pandemic cohorts. A statistically significant difference has been observed between pre-pandemic and mid-pandemic populations (*p* < 0.0001) and between mid-pandemic and COVID19 populations (*p* < 0.0001). No significant difference has been found in COVID19 group compared with the pre-pandemic population (Figure 5).

From these results, 11 COVID19 patients were selected based on the plasma antibody response against the COV2-SPIKE_421–434_ peptide (five strong and six medium responders). All the strongly responsive patients showed outstanding antibody cross-reactivity with a noticeable reduction of the antibody reaction after the incubation with NL63-RBM3 peptide.

**Figure 6:**
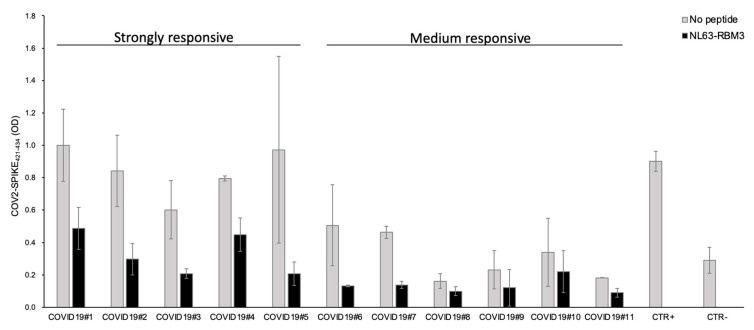
Competition assay with COV2-SPIKE_421–434_ coated ELISA plate. Plasma from 11 COVID19 patients, with strong and medium antibody response against COV2-SPIKE_421–434_ epitope, were pre-incubated overnight with NL63-RBM3. The grey and black bars represent plasma reactivity against COV2-SPIKE_421–434_ under normal conditions and after incubation with NL63-RBM3, respectively. Positive and negative controls were included in the assay.

Particularly, the competition ELISA showed a reduction of 51.2% for COVID19#1, 64.8% for COVID19#2, 65.4% for COVID19#3, 43.7% for COVID19#4 and 78.6% for COVID19#5. The plasma from medium responsive patients showed a reduction of the antibody response of 73.9% for COVID19#6, 70.1% for COVID19#7, 38.6% for COVID19#8, 47.6% for COVID19#9, 35.1% for COVID19#10, 50.9% for COVID19#11 (Figure 6).

## 4. Discussion

Cross-reactivity between HCoVs has long been hypothesized to offer transient protection against infection with different CoVs [7,18]. In particular, Sagar et al. [19] have reported less severe coronavirus disease 2019 illness in patients with a previously detected endemic human CoVs (eCoV) infections; they suggested that pre-existing immune responses against eCoVs can alleviate disease manifestations from SARS-CoV-2 infection.

Three known antigenic groups of CoVs are associated with diseases in animals and humans. The known human CoVs (HCoVs) are HCoV- 229E and HCoV-NL63 belonging to the alpha group and HCoV-OC43 within the beta group, are generally recognized to cause mild upper respiratory tract diseases and, rarely, lower respiratory tract diseases. However, HCoV-NL63 is the only CoV responsible of mild infections that use the ACE2 receptor as target for its S protein [20].

HCoV-NL63 S-protein has been well characterized. Although HCoV-NL63 and SARS-CoV have no structural homology in RBD cores or RBM, the two viruses recognize common ACE2 regions, largely because of a “virus-binding hotspot” on ACE2. Using the IEDB site we tried to identify possible common “binding hotspot” on ACE2 by analyzing the S-proteins on both CoVs. Moreover, S-protein structural and phylogenetic analysis revealed low homology in the aminoacidic sequence in SARS-CoV and SARS-CoV-2, on the other hand both S-protein RBDs manifested the same ACE2-binding mode [21].

With regard to what expressed above, we wondered if a serologic cross-reactivity may be found between HCoV-NL63 and SARS-CoV-2. To date, few studies investigated antibody cross-reaction between alpha and beta-coronaviruses.

Recently, Loos et al. [22] reported some positive relationships between the IgGs response to the common CoVs and SARS-CoV-2 RBD-specific immunity, most relationships among the SARS-CoV-2 response and common CoVs, influenza virus, and Respiratory Syncytial Virus (RSV) were largely driven by individuals exhibiting low to undetectable titers. They suggest that preexisting CoV RBD-specific and other pathogen immunity plays a limited role in shaping SARS-CoV-2 humoral immune responses. However, they used the HCoV-NL63 RBD (accession no. AKT07952, aa residues 481 to 616) without any immunogenic analysis of selected highly immunogenic B cells epitopes as we performed.

Recently, three well designed studies were published in Nature, Science and Cell journals, showing evidence of cross-reactive T-cell immunity between human coronaviruses (229E, NL63, OC43, and HKU1), and SARS-CoV-2 [23,24,25].

Mateus et al. [25] showed three cases in which HCoV analogs were better antigens than the SARS-CoV-2 peptide, suggesting that they may be the cognate immunogen, one of them belonging to HCoV-NL63.

These findings of cross-reactive HCoV T-cell specificities are in stark contrast to HCoV-neutralizing antibodies, which are HCoV species specific and did not show cross-reactivity against SARS-CoV-2 RBD as reported by Premkumar et al. [26]. Here, archived human samples collected before SARS-CoV-2 (20 American adults and samples from individuals in South Asia, the Caribbean, and Central America) were tested for binding against RBD spike antigens from HCoVα (NL63) and HCoVβ (HKU1) with no reaction [27]. Whereas Sotgia F. and Lisanti MP. argued that mild pathogenic coronaviruses epitopes can be used for a vaccine [26].

Of note, Ng et al. (Science, 2020) have identified several epitopes that were recognized with cross-reactive antibodies by uninfected patients proving the presence of preexisting antibodies recognizing SARS-CoV-2 [28]. These results considering also the finding of preexisting T cell memory against seasonal HCoVs and SARS-CoV-2 (21) may shed light on the SARS-CoV-2 natural infection having in mind that this protective cross immunity does not last for long time and probably is not sterilizing as well.

In our study, we first assessed the antibody response against five peptides of HCoV-NL63 and for two epitopes of SARS-CoV-2 from S-protein in a pre-pandemic and a mid-pandemic group. The pre-pandemic population shows good chances to have been recent infected with eCoVs, including NL63, as it is composed for about one-third of patients under 18 years old, that are the most exposed people [29]. The decision to use short peptides, instead of expressed protein, represents a limitation of the study, but it can also a resource showing epitopes that could be previously hidden.

The pre-pandemic population exhibited a strong humoral response against HCoV-NL63 compared to that observed in the mid-pandemic population for NL63-RBM1 and NL63-DISC-like.

At the same time, we observed an unexpected and strong humoral response in the pre-pandemic population towards SARS-CoV-2 S-protein epitopes. Of course, this response cannot be attributed to SARS-CoV-2 since the samples from the pre-pandemic population were collected before 2019. These data were confirmed by the rapid test we performed on one of the most reactive pre-pandemic sample for SARS-CoV-2 epitopes, obtaining a positive result. Considering that 72.5% and 68.7% of the pre-pandemic population are seropositive against COV2-SPIKE_421–434_ and COV2-SPIKE_742–759_, respectively, we think a heterologous cross-reactivity between HCoV-NL63 antibodies against SARS-CoV-2 epitopes.

The correlations found between HCoV-NL63 and SARS-CoV-2 peptides in both cohorts corroborate the hypothesis of an antibody cross-reactivity between the two virus species and the possibility that HCoV-NL63 may offer a certain degree of protection against SARS-CoV-2. Further, we have looked at the correlations among the epitopes used in this study in pre-pandemic population divided by age classes (Figure 4); the finest correlation can be observed in 5–10 years group and in 40–60 years group. Of course, to better understand this correlation more in depth-studies are necessary but one of the assumptions can be that the antibodies against NL63 may help for a better manage of the SARS-CoV-2 infection.

To confirm these data, the results from a cELISA, performed on patients with a positive diagnosis to SARS-CoV-2, highlight a significant difference in antibody response against SARS-CoV-2 epitopes between pre-pandemic and mid-pandemic cohorts and between COVID19 and mid-pandemic groups. Noteworthy, HCoV-NL63 infections were observed primarily in the winter season, period when our pre-pandemic samples were collected with a 30.6% of children under 18 years old, that represent the most HCoV-NL63 affected people [29]; this can explain the similar antibody response in pre-pandemic and COVID-19 groups. Related to this, Canducci et al. demonstrated that on 322 infants suffering from acute respiratory disease, the 8.7% of the cases examined were caused by coronaviruses, with HCoV-NL63 accounting for 21.4% of the latter [30].

This support our initial hypothesis that antibodies directed against HCoV-NL63 S-protein may recognize SARS-CoV-2 S-protein epitopes.

These results are particularly relevant in both strongly and medium responsive patients (Figure 6), we observed a significant reduction of the antibodies against COV2-SPIKE_421–434,_ except for two patients (COVID19#8 and COVID19#10) who showed a reduction inferior to 40%. Considered the increased seroprevalence of HCoV-NL63 infections and the low incidence of SARS-CoV-2 in children, it is possible that antibodies directed against HCoV-NL63 may have a protective effect in some individuals affected by SARS-CoV-2.

A richer interaction energies of the human ACE2 molecular recognition by SARS-CoV-2 compared to SARS-CoV and HCoV-NL63 was recently reported [31], explaining why SARS-CoV-2 has an enhanced ability for pathogenicity. An other study [32] identified the conserved residues in the binding interface of S proteins in all three strains. The systematic point mutations show that these conserved residues in the respective strains are evolutionarily favored at their respective positions. Notwithstanding, further experiments, larger cohorts and different time collection, are necessary to establish the length of the protection and to assess the nature of the antibodies and their possible neutralizing effect against SARS-CoV-2, but also to investigate the possible contribution of pre-existing immunity offered from other coronaviruses, such as HCoV-OC43, mediated by different proteins than Spike.

## Figures and Tables

**Table 1:** Details of pre-pandemic, mid-pandemic, and COVID-19 patients samples used in this study.

	Pre-Pandemic Patients (*n* = 160)	Co-Pandemic Patients (*n* = 141)	COVID-19 Patients (*n* = 46)	
			ICU (*n* = 4)	Other Clinical dpt.(*n* = 42)
**Age** **(mean ± SD)**	39.63 ± 22.85	55.55 ± 16.99	72.5 ± 4.36	56.95 ± 17.46
**Gender** **(male/female)**	51/109	36/105	3/1	17/25

**Table 2:** Epitopes identified in HCoV-NL63 and SARS-CoV-2.

	Epitope Position	Epitope Sequence
NL63-RBM1	S_433–446_	FGGSCYVCKPHQVNI
NL63-RBM2_1	S_469–482_	NRVKSGSPGDSSWH
NL63-RBM2_2	S_471–481_	VKSGSPGDSSW
NL63-RBM3	S_525–539_	WHYTSYTIVGALYVT
NL63-SPIKE_541–554_	S_541–554_	SEGNSITGVPYPVS
NL63-DISC-like	S_58–78_	SGGRGSGRGGNLTYLNLSSEL
COV2-SPIKE_421–434_	S_421–434_	FSQILPDPSKPSKRSFIE
COV2-SPIKE_742–759_	S_742–759_	CNGVEGFNCYFPLQS

**Table 3:** Details of pre-pandemic population divided by age groups (years old, y/o)

Age Groups	Number of Patients
**5–10 y/o**	14
**11–20 y/o**	33
**21–40 y/o**	25
**41–60 y/o**	50
**61–80 y/o**	35
